# Prevalence and Etiology of Diarrhea in Children Under 5 Years Old at the Ngaoundere Regional Hospital, Cameroon: A Cross‐Sectional Study

**DOI:** 10.1002/hsr2.71381

**Published:** 2025-10-15

**Authors:** Michel Archange Fokam Tagne, Benjamin Talom Tangue, Emilie Laurence Kuete Diffo, Paul Aimé Noubissi, Angèle Foyet Fondjo, Yafet Dourkangou, René Kamgang

**Affiliations:** ^1^ Department of Biological Sciences, Faculty of Science University of Ngaoundere Cameroon; ^2^ Department of Biomedical Sciences, Faculty of Science University of Ngaoundere Cameroon; ^3^ Department of Zoology and Animal Physiology, Faculty of Science University of Buea Cameroon; ^4^ Department of Applied Sciences for Health, Higher Institute of Applied Sciences University Institute of Gulf of Guinea Cameroon; ^5^ Department of Geography, Geomatics Laboratory University of Ngaoundere Cameroon; ^6^ Laboratory of Endocrinology and Radioisotopes Institute of Medical Research and Medicinal Plants Studies (IMPM) Yaoundé Cameroon

**Keywords:** bacteria, children under 5 years old, diarrhea, Ngaoundere regional hospital, parasites

## Abstract

**Background and Aims:**

Diarrhea is a worldwide public health problem in children under 5 years old. This study aimed to determine the main etiology of diarrhea affecting children under 5 years old at the Ngaoundere Regional Hospital.

**Methods:**

The present study was a cross‐sectional survey of patients under 5 years of age with diarrhea which was conducted at the Ngaoundere Regional Hospital from May to August 2023. With the use of a questionnaire, a total of 179 patients aged under 5 years with diarrhea were sampled. A questionnaire was submitted to the patients to collect socio‐demographic information then stool samples were collected. The stool samples were then subjected to the macroscopic and microscopic analyses phases for the detection of parasites, and to coproculture or stool culture for the subsequent isolation of the pathogenic bacterial germs. The identification of bacterial germs was carried out using the API20E gallery.

**Results:**

Male subjects were most affected by diarrheal diseases with 57% compared to 43% for females and the age group (12–24 months) was the most vulnerable (31.84%). Among the patients, 37.43% had a yeast infection, 24.58% a parasitic infection, and 23.58% a bacterial infection. *Entamoeba histolytica* (15.64%), *Entamoeba coli* (6.70%), *Trichomonas intestinalis* (2.23%), and *Fasciola hepatica* (0.56%) were identified as parasites. *Escherichia coli* (10.61%), *Salmonella spp*. (5.02%), *Klebsiella spp*. (3.35%), *Klebsiella pneumoniae* (1.12%), *Shigella spp.* (1.12%), *Enterobacter cloacae* (1.12%), *Enterobacter aerogenes* (0.56%), *Enterobacter spp*. (0.56%), *Hafnia alvei* (0.56%) and *Pseudomonas spp*. (0.56%) were identified as bacteria. 2.79% cases of parasitic/bacterial co‐infections were recorded.

**Conclusions:**

The prevalence of diarrhea caused by parasites and bacteria in children under 5 years of age at the Ngaoundere Regional Hospital remains high compared to the prevalence of diarrhea in Cameroon. The most incriminated infectious agents were *Entamoeba histolytica* and *Escherichia coli*. These etiological data would constitute important tools for strategies for the management and prevention of childhood diarrhea.

## 1 Introduction

Infectious diarrhea is generally a symptom of an intestinal tract infection due to pathogens such as bacteria, parasites and/or viruses [1]. According to the World Health Organization, diarrhea is the second leading cause of death among children under the age of five, with 1.7 billion cases and approximately 525,000 deaths each year worldwide. Diarrhea is defined as the passing of at least three loose or liquid stools in 24 h [1], and is among the most frequent and widespread diseases in the world [2]. Despite overall improvements in water quality, sanitation, and hygiene over the decades, infectious diarrhea remains a significant cause of mortality and morbidity for children under 5 years of age worldwide, particularly in developing countries [3, 4]. This would be due to difficult access to quality health care and late treatment [5, 6]. In developing countries, a child presents on average 3–9 diarrheal episodes per year and this pathology represents the primary reason for hospitalization in pediatric settings [7, 8]. According to a statistical report provided by UNICEF, sub‐Saharan Africa alone records 30% of overall deaths due to diarrhea among children under 5 years old worldwide [9].

In Cameroon, diarrhea is the main cause of infant morbidity and mortality among children under 5 years after acute respiratory infections [10]. The prevalence varies from one region to another: 10.4% in Adamawa compared to 12.8% in the south of the country [11]. Another study in Yaoundé (Cameroon) showed that out of 437 patients with diarrhea, 260 (59.5%) had an infectious cause. Among the microorganisms associated with childhood infectious diarrhea, viruses, bacteria, and parasites presented 3.8%, 36.9%, and 59.2% of the cases, respectively [12].

Children with diarrhea are at risk for many health issues, including loss of appetite, electrolyte deficiency, malnutrition, increased risk of developing other infectious diseases, and delayed physical growth and mental development [13]. Although treatment of diarrhea with rehydration solutions (ORS) is improving in terms of global coverage, ORS coverage rates are still far too low in central and west Africa [9]. As a result, diarrhea continues to be a public health problem, especially in Cameroon where it is endemic. However, the scientific documentation on infectious diarrhea remains very poor in Cameroon in all aspects and the management is based in most cases on clinical arguments and a presumptive diagnosis [14]. In a case study in Yaounde, Cameroon, out of 437 patients under 5 years of age with diarrhea, 260 (59.5%) were of infectious etiology. Among these cases, 59.2%, 36.9% and 03.8% were of parasitic, bacterial and viral origin, respectively. Among the pathogens, the most frequently found were *Ascaris lumbricoides* (17.8%), *Giardia lamblia* (13.2%), *Trichuris trichiura* (10.7%), *Salmonella spp*. (9.6%), *Campylobacter spp*. (8.8%), Entamoeba (8.4%), *Shigella spp*. (7.3%), *Esherichia coli*—enteropathogenic (4.2%) and Enteric adenovirus (2.7%) [12]. Early diagnosis of specific causal agents of diarrhea allows the disease to be better resolved and prevents a second infection [15]. Epidemiological data on infectious diarrhea (prevalence and causal agents) in the locality of Ngaoundere (Cameroon) are rare, hence the need to survey and identify the microorganisms associated with diarrhea in the locality of Ngaoundere. The main objective of this study was to determine the main causes of infectious diarrhea in the locality of Ngaoundere to improve its management.

## 2 Materials and Methods

### 2.1 Study Location

**Figure 1:**
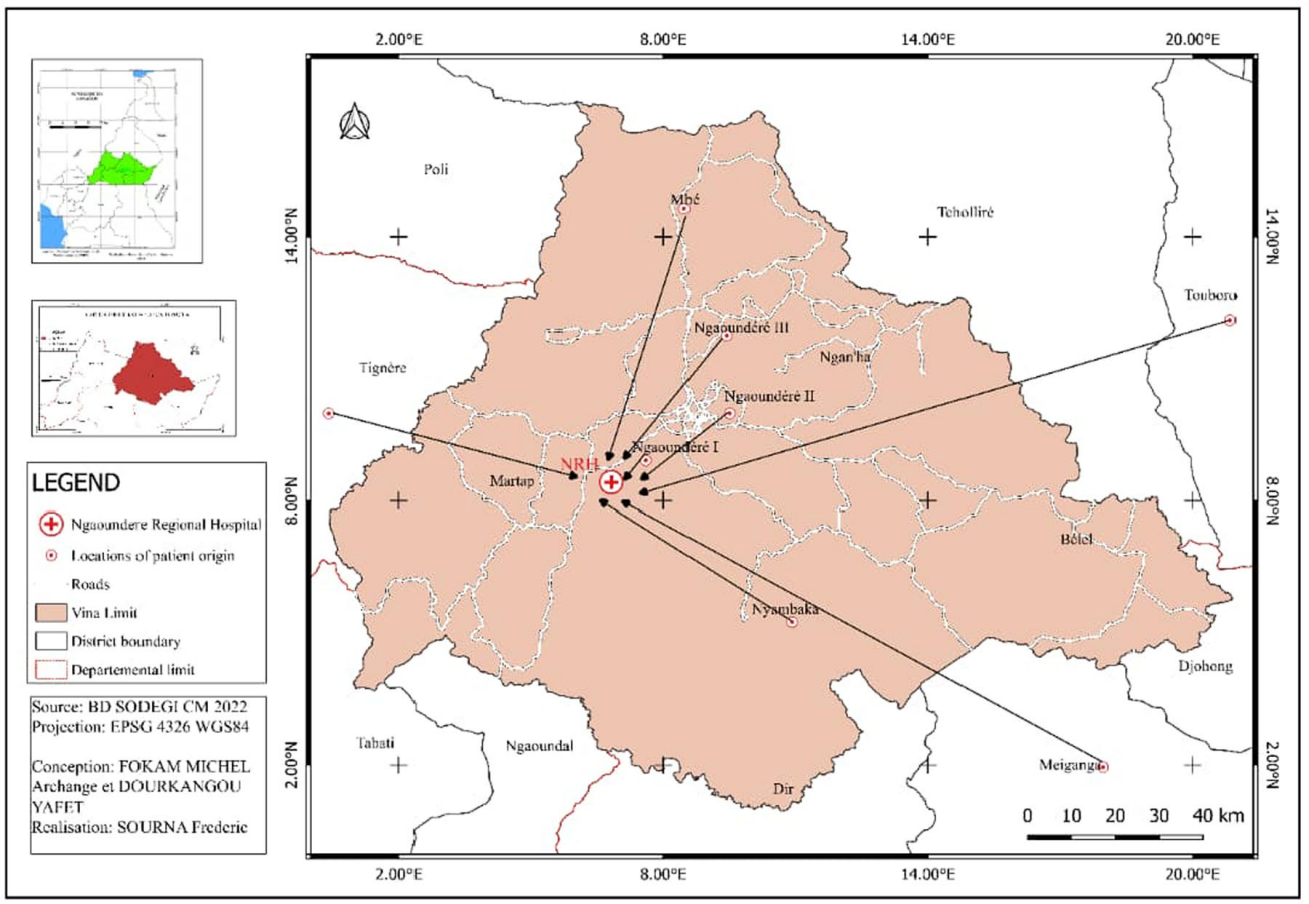
Location map of the Ngaoundere Regional Hospital (NRH) and the places of patient's origin.

The study took place at the Ngaoundere Regional Hospital (NRH) in the pediatric department from May 3, 2023 to August 26, 2023. NRH is located in the Ngaoundere I district, Vina Department, Adamawa Region (Cameroon), situated between latitudes 7°0′ and 8°0′ N and 13°0′ and 14°0′ E (Figure 1). The climate is humid Sudano‐Guinean type, with average annual rainfall of approximately 1500 mm per annum. The population of the Adamawa region is cosmopolitan, with approximately 1,015,622 inhabitants, or 15.9 inhabitants per km². Livestock farming remains the main economic activity, practiced by more than 20% of the rural population [16].

NRH is a third category in the Cameroonian health system and second reference hospital establishment in the Adamawa Region. The NRH is made up of a management and 17 services including the Reception and Emergency, Internal Medicine, Ophthalmology, Odontostomatology, Intensive Care, Pharmacy, Pediatrics, and Laboratory services. The NRH pediatrics department has six units which are: neonatology, general pediatrics, the internal therapeutic nutrition center (ITNC), vaccination, the center for the care of diabetic children, and the center for the care of children living with HIV/AIDS. This department has a capacity of 55 hospitalization beds. It carries out several activities including outpatient consultation, hospitalization and daily monitoring of patients, continuing training of staff, and supervision of trainees.

### 2.2 Patients and Study Design

The study was conducted on 179 patients aged under 5 years old, who were admitted to the Ngaoundere Regional Hospital in the pediatric department from May 3, 2023 to August 26, 2023. The sample size was calculated from the Lorentz formula using a margin of error set at 5%, at the confidence level *t* = 1.96 taking into account the prevalence of childhood diarrhea in Cameroon which was 12% in 2018 [17, 18].

$$n=\frac{\left(z^{2}\right)P\left(1-P\right)}{d^{2}}$$

With: *n *= sample size; *P *= prevalence of childhood diarrhea in Cameroon (12%); *z* = 1.96 confidence level; and *d *= desired precision with error of 0.05.

According to the World Health Organization, diarrhea is defined as at least three loose stools in 24 h with at least one of the following symptoms: nausea, vomiting, abdominal cramps, with or without fever [19].

Inclusion criteria were all children aged 0–59 months suffering from diarrhea, consulting the Ngaoundere Regional Hospital and whose parents had accepted assent. Exclusion criteria were children over 5 years of age, children under 5 years of age with malabsorption, immunosuppression, and children under 5 years of age on immunosuppressive therapy or prolonged steroid therapy. Exclusion criteria were children over 5 years of age, children under 5 years of age with malabsorption, immunosuppression, and children under 5 years of age on immunosuppressive therapy or prolonged steroid treatment. Indeed, malnutrition, immunosuppression, immunosuppressants, and steroids could be the cause of diarrhea [20]. After applying the inclusion and exclusion criteria, all eligible patients were selected and stool samples were collected.

### 2.3 Data Collection

Stool samples were collected on the days of admission in sterile vials labeled with the date and time of collection, the name, gender, and age of the patient, and sent directly to the laboratory. An information sheet in the form of structured questionnaires made it possible to collect information relating to the socio‐demographic characteristics (gender, age, and residence) of the patients and the appearance of the stools (color, consistency, microbiological aspect).

For macroscopic examinations of the stools, the color (greenish, yellowish, brown, etc.), the consistency (liquid, soft, molded, pasty, etc.) of the stools as well as the presence of mucus, pus and/or blood on the stools were noted. Hematophagous forms of amoebae were also sought in the mucus and presence of certain adult parasites (Ascaris, pinworm, tapeworm ring). Identification of parasites in stool samples was done after staining slides with chlorazol black and microscopic observation [21, 22].

For microscopic examinations, cytological and bacteriological studies were performed. The cytology study was carried out by direct observation in the fresh state under a microscope (at ×10 and ×40 magnification) of the following elements: leukocytes, yeasts, red blood cells, epithelial cells, monocrystalline cells, and potential parasites (*Entamoeba histolytica, Giardia lamblia, Trichomonas intestinalis*).

**Figure 2:**
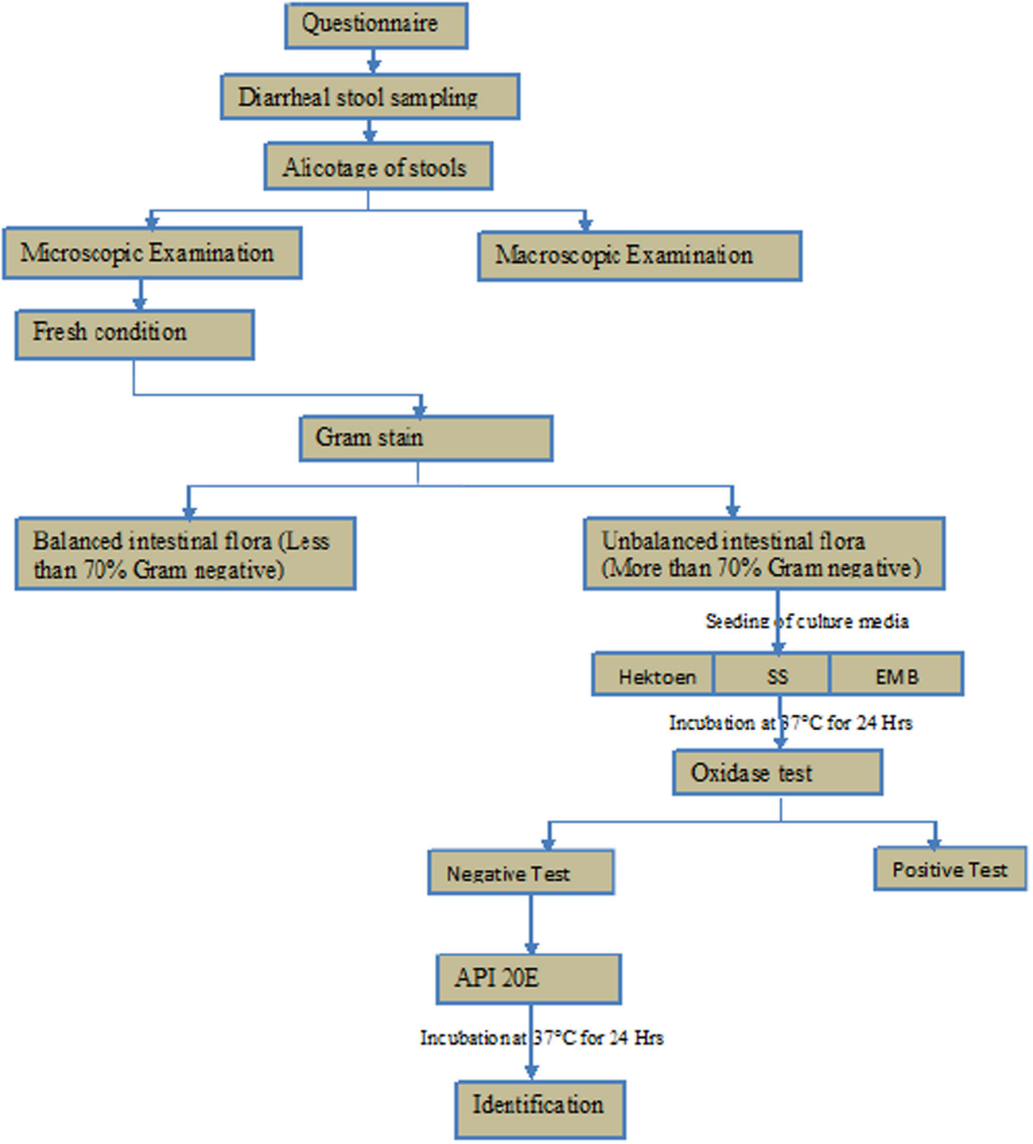
Block diagram of the research and identification of bacteria.

The bacteriological study consisted of observation under a microscope after the Gram staining. This study firstly allows the balance between Gram‐positive and Gram‐negative bacteria in the intestinal flora to be assessed. A flora was said to be balanced if it consisted of approximately 70% Gram‐negative bacilli, and unbalanced if it consisted of more than 70% Gram‐negative bacteria. The unbalanced flora was then inoculated using a sterilized platinum loop in culture media, Hoektoen medium, Salmonella/Shigella (SS) medium, and EMB and incubated for 24 h at 37°C. After 24 h of incubation, oxidase tests were performed on isolated pure colonies. The oxidase‐positive germs were rejected. The oxidase‐negative germs were identified by standard microbiological methods and API tests [23] (Figure 2).

### 2.4 Data Analysis

The sociodemographic variables analyzed were the patients' sex, age and place of residence. Regarding stool appearance, the variables were color, consistency, and the presence of mucus, leukocytes, yeasts, red blood cells, pathogenic bacteria and potentially pathogenic parasites. The data were classified and entered into Microsoft Excel 2016 software (Microsoft Corporation) and then exported to GraphPad Prism version 8.0.1 software (Crunchbase Company) for data analysis. The distribution of patients consulted was presented as frequencies and percentages per variable. The Pearson *χ* ^2^ test was used to test the association of each independent variable with another data variable. A measure of fit of the multivariate ordinal logit model to the data was verified using a likelihood ratio *χ* ^2^ test at a *p* value < 0.05 with 95% confidence intervals for the significance of the predictor variables relative to the outcome variable.

### 2.5 Research Ethics Approval and Informed Consent

For this study, approval was obtained from the Adamawa Regional Health Delegation under number No. 452/L/RA/DSP/SAG/NGD on May 26, 2023. Informed assent form was obtained from the children's parents.

## 3 Results

### 3.1 Socio‐Demographic Characteristics of Patients

The patients surveyed in this study came from eight localities in the Adamawa Region: Ngaoundere‐1 108 (60.33%), Ngaoundere‐2 59 (32.96%), Ngaoundere‐3 3 (1.67%), Touboro 3 (1.67%), Mbe 2 (1.10%), Nyambaka 2 (1.11%), Meiganga 1 (0.55%) and Tignere 1 (0.55%). Among the total study participants, 102 (56.98%) were males, while 77 (43.02%) were females, a sex ratio of 1.3. The most marked age range was 12–23 months of age with a total of 57 (31.84%) (Table 1).

**Table 1:** Frequency of patients according to place of origin, sex, and age.

Origin	Sex	Age (month)	*N*	%	Total	Origin	Sex	Age (month)	*N*	%	Total
Mbe	Males	[0–6]	0	0	1 (0.56)	Ngaoundere‐3	Males	[0–6]	1	0.56	3 (1.68)
		[6–12]	0	0				[6–12]	0	0	
		[12–24]	0	0				[12–24]	0	0	
		[24–36]	0	0				[24–36]	0	0	
		[36–48]	0	0				[36–48]	2	1.12	
		[48–60]	1	0.56				[48–60]	0	0	
	Females	[0–6]	0	0	1 (0.56)		Females	[0–6]	0	0	1 (0.56)
		[6–12]	0	0				[6–12]	0	0	
		[12–24]	0	0				[12–24]	0	0	
		[24–36]	1	0.56				[24–36]	0	0	
		[36–48]	0	0				[36–48]	0	0	
		[48–60]	0	0				[48–60]	0	0	
Meiganga	Males	[0–6]	0	0	0 (0.00)	Nyambaka	Males	[0–6]	0	0	1 (0.56)
		[6–12]	0	0				[6–12]	0	0	
		[12–24]	0	0				[12–24]	0	0	
		[24–36]	0	0				[24–36]	1	0.56	
		[36–48]	0	0				[36–48]	0	0	
		[48–60]	0	0				[48–60]	0	0	
	Females	[0–6]	0	0	1 (0.56)		Females	[0–6]	0	0	1 (0.56)
		[6–12]	0	0				[6–12]	0	0	
		[12–24]	0	0				[12–24]	0	0	
		[24–36]	1	0.56				[24–36]	0	0	
		[36–48]	0	0				[36–48]	1	0.56	
		[48–60]	0	0				[48–60]	0	0	
Ngaoundere‐1	Males	[0–6]	10	5.58	60 (33.52)	Tignere	Males	[0–6]	0	0	0 (0.00)
		[6–12]	10	5.58				[6–12]	0	0	
		[12–24]	20	11.17				[12–24]	0	0	
		[24–36]	10	5.58				[24–36]	0	0	
		[36–48]	1	0.56				[36–48]	0	0	
		[48–60]	9	5.03				[48–60]	0	0	
	Females	[0–6]	9	5.03	48 (26.81)		Females	[0–6]	0	0	1 (0.56)
		[6–12]	8	4.47				[6–12]	0	0	
		[12–24]	20	11.17				[12–24]	1	0.56	
		[24–36]	7	3.91				[24–36]	0	0	
		[36–48]	1	0.56				[36–48]	0	0	
		[48–60]	3	1.67				[48–60]	0	0	
Ngaoundere‐2	Males	[0–6]	5	2.79	35 (19.55)	Touboro	Males	[0–6]	0	0	2 (1.12)
		[6–12]	7	3.91				[6–12]	0	0	
		[12–24]	8	4.47				[12–24]	0	0	
		[24–36]	6	3.35				[24–36]	0	0	
		[36–48]	5	2.79				[36–48]	2	1.12	
		[48–60]	4	2.23				[48–60]	0	0	
	Females	[0–6]	2	1.12	24 (13.41)		Females	[0–6]	0	0	1 (0.56)
		[6–12]	3	1.67				[6–12]	0	0	
		[12–24]	7	3.91				[12–24]	0	0	
		[24–36]	4	2.23				[24–36]	1	0.56	
		[36–48]	4	2.23				[36–48]	0	0	
		[48–60]	4	2.23				[48–60]	0	0	

### 3.2 Macroscopic and Microscopic Appearance of Stools

The stools sampled were mostly soft (63.68%), heterogeneous (74.30%) and yellowish in color (50.27%). The proportion of stools that presented mucus was 17.31%. Microscopic analysis of stools sampled from patients showed a high density of leukocytes (64.80%) and a low density of red blood cells (3.91%) (Table 2).

**Table 2:** Macroscopic and microscopic appearance of stools.

Appearance	Variable		Effective	Frequency (%)
Macroscopic appearance	Consistency of stools	Watery	36	20.11
		Soft	144	63.68
		Pasty	89	16.20
	Stool color	Yellowish	90	50.27
		Whitish	1	0.56
		Greenish	66	36.87
		Greyish	4	2.23
		Brown	17	9.50
		Reddish	1	0.56
	Appearance of stools	Heterogeneous	133	74.30
		Homogeneous	46	25.70
	Mucus	Presence	31	17.31
		Absence	148	82.68
Microscopic appearance	Red blood cells	Presence	7	3.91
		Absence	172	96.09
	Leukocytes	Presence	116	64.80
		Absence	63	35.20

### 3.3 Main Groups of Infectious Agents Identified

From May 2023 to August 2023, the total proportions of infectious agents were 37.43%, 24.58%, and 24.02% respectively for yeasts, parasites, and bacteria (Table 3). Cases of parasite/bacteria co‐infections were observed in 2.79% of the cases. This included co‐infection between the parasitic germ *Entamoeba histolytica* and the bacterial genera *Escherichia coli* (1.12%), *Klebsiella spp*. (1.12%) and *Enterobacter spp*. (0.56%).

**Table 3:** Monthly proportion of pathogens responsible for diarrhea recorded in Ngaoundere Regional Hospital from May 2023 to August 2023.

Month	*N* (%)	Proportion (%)			*χ* ^*2*^	Df	*p* value
		Parasites	Bacteria	Yeasts			
May	67 (37.43%)	6.68^*a*^	3.90^*a*^	4.60^*b*^	67.60	2	*p* < 0.001
June	29 (16.20%)	6.70^*ab*^	2.22^*a*^	4.43^*b*^	16.42	2	*p* < 0.001
July	32 (17.88%)	5.60^*ab*^	4.50^*a*^	3.80^*b*^	10.51	2	*p* < 0.005
August	50 (27.93%)	5.60^*a*^	13.40^*b*^	24.60^*c*^	26.28	2	*p* < 0.001
Total	179 (100%)	24.58	24.02	37.43			

*Note:* The proportions of infectious agents from the same line (month), and assigned the same letters (a, b, and c) do not show any difference at the risk threshold *α* = 5%. *χ* ^2 ^= Chi‐square, Df = degree of freedom, *p *= probability.

### 3.4 Frequency of Parasitic Diarrhea and Bacterial Diarrhea According to Age Group and Sex

The frequency of parasitic infections was higher in the age groups (12–24) (7.82%), [24–36] (7.82%), and (48–60) (4.46%) months. As for bacterial infections, the age group (12–24) months was the most infected with a frequency of 9.48% followed by the age group (0–6) with a frequency of 4.46% (Table 4).

**Table 4:** Frequency of patients consulted, parasitic diarrhea and bacterial diarrhea according to age group and sex.

	Patients consulted (%)			Parasitic diarrhea (%)			Bacterial diarrhea (%)		
Age (month)	Males	Females	Total	Males	Females	Total	Males	Females	Total
[0–6]	8.38	6.15	14.53	0.56	0.56	1.12	2.23	2.23	4.47
[6–12]	9.50	6.15	15.64	1.12	1.12	2.23	2.23	0.56	2.79
[12–24]	16.20	15.64	31.84	3.91	3.91	7.82	4.47	5.03	9.50
[24–36]	10.61	7.82	18.44	4.47	3.35	7.82	1.68	1.68	3.35
[36–48]	4.47	3.35	7.82	1.12	0.00	1.12	1.12	0.56	1.68
[48–60]	7.82	3.91	11.73	3.35	1.12	4.47	1.12	0.56	1.68
Total	56.98	43.02	100	14.53	10.06	24.58	12.85	10.62	23.47
Sex ratio (M/F)	1.30			1.44			1.21		

### 3.5 Prevalence of Infectious Agents Identified in Stool Samples

Four parasitic species were identified in the stool samples: *Entamoeba histolytica* (15.64%), *Entamoeba coli* (6.70%), *Trichomonas intestinalis* (2.23%), and *Fasciola hepatica* (0.56%).

Bacterial analyses of stools revealed ten (10) potentially infectious bacterial strains: *Enterobacter aerogenes* (0.56%), *Enterobacter cloacae* (1.12%), *Enterobacter spp.* (0.56%), *Escherichia coli* (10.61%), *Hafnia alvei* (0.56%), *Klebsiella pneumoniae* (1.12%), *Klebellsia spp.* (3.35%), *Pseudomonas spp.* (0.56%), *Salmonella spp.* (5.02%), and *Shigella spp.* (1.12%). The *χ* ^2^ test of independence between the presence of leukocytes and those of infectious agents shows that the presence of infectious bacteria in the stools is correlated with the presence of leukocytes (*χ* ^2^: 27.054; *p*: 0.005). However, the presence of parasites and yeasts in the stools is not correlated with the presence of leukocytes (Table 5).

**Table 5:** Test of independence of *χ* ^2^ between the presence of leukocytes and that of infectious agents.

Variable		Effective	Frequency (%)	Df	*χ* ^*2*^	*p* value
Parasites	Absent	135	75.42	5	2.399	0.792
	*Entamoeba histolytica*	28	15.64			
	*Entamoeba coli*	12	6.70			
	*Fasciola hepatica*	1	0.56			
	*Trichomonas intestinalis*	4	2.23			
Bacteria	Absent	136	75.98	11	27.054	0.005
	*Salmonella spp.*	9	5.03			
	*Shigella spp.*	2	1.12			
	*Klebsiella spp.*	6	3.35			
	*Klebsiella pneumoniae*	2	1.18			
	*Enterobacter cloacae*	2	1.18			
	*Enterobacter spp.*	1	0.56			
	*Enterobacter aerogenes*	1	0.56			
	*Hafnia alvei*	1	0.56			
	*Escherichia coli*	19	10.62			
	*Pseudomonas spp.*	1	0.56			
Yeasts	Absent	34	18.99	2	0.708	0.702
	Non‐pathogenic	78	43.58			
	Pathogenic	67	37.43			

Abbreviations: *χ* ^2^ = Chi‐square, Df *= *degree of freedom, *p* = probability.

## 4 Discussion

Infectious diarrhea is generally a symptom of an intestinal tract infection caused by pathogens such as bacteria, parasites and/or viruses [1]. These are most often acute and correspond to too abundant and too frequent emissions of stools, which appear suddenly with a spontaneous resolution within a few days (less than 14 days) [24]. Despite overall improvements in water quality, sanitation, and hygiene over the decades, infectious diarrhea remains a significant cause of mortality and morbidity for children under 5 years of age worldwide, particularly in developing countries [3, 4].

This study was carried out over a 4‐month period and aimed to identify pathogens in stool samples collected from 179 children aged 0–60 months, admitted at the Ngaoundere Regional Hospital. Patients from the Ngaoundere I district were the most represented, which could be explained by the fact that the Ngaoundere Regional Hospital is located in this Ngaoundere I District. Regarding the age, the most affected patients were aged between 12 and 23 months. A study carried out in Ethiopia on the prevalence of diarrhea showed that children aged between 12 and 23 months were 4.1 times more affected by diarrhea [25]. Another similar study carried out in the city of Yaoundé (Cameroon), also showed that children in this age group were more affected (40.73%) by infectious diarrhea [12]. The vulnerability of this age group to enteric infections could be explained by the modification of the child's diet which becomes much more diversified from 12 months, and/or by the immaturity of the intestinal flora and the child immune system at this age. This vulnerability could also be explained by the fact that during this period of life, children tend to explore the environment and bring everything they can have access to, into their mouths. Furthermore, the male sex was more prone to diarrheal episodes with a sex ratio of 1.3. This value is close to that reported in studies on the pathogens responsible for enteric infection in Burkina [26], Mali [27], and Cameroon [12]. Some authors indicate that the predilection for the male sex could be explained by genetic and immunological factors, making them more susceptible to the development of severe forms requiring hospitalization [28].

The majority of stools were soft, yellowish in color, and heterogeneous in appearance with the presence of mucus and/or blood. These results and corroborate those reported in Mali [27] and partially those reported by a study in Burkina Faso in which the predominance was rather linked to liquid diarrhea with nevertheless a low proportion of mucus found in the stools [26]. Microscopic analysis of the stool samples revealed that a small proportion was associated with the presence of red blood cells, unlike leukocytes which were detected at a high frequency in the stools. The presence of red blood cells and/or leukocytes in diarrheal stools is most often due to intestinal infections by invasive germs such as *Salmonella*, *Shigella*, *Entamoeba histolytica*, or even entero‐invasive *Escherichia coli* [29].

The infectious causes of diarrhea in patients between May 2023 and August 2023 were fungal, parasitic, and bacterial; a viral origin could be attributed to the 44% of cases of liquid diarrhea in which no infectious germ was identified. The frequency of parasites in stool samples was close to that obtained in a study on parasitic diarrhea (25.6%) in Nairobi, Kenya [30], and differed from those obtained in other studies of the same type in Burkina Faso [31], in Port Blair, Andaman and Nicobar Islands [32], and in Yaoundé, Cameroon [12] with 59.23%, 18.80% and 57.80% respectively. This could be explained by the differences that exist in the methodology of the different studies carried out, in particular the period and duration of the study; some taking place over an entire year and others over several seasons. In addition, the observed difference could also be explained by the diversity of geographical sites and the age of previous studies given the various improvements made in terms of drinking water supply and sanitation facilities. Microscopic analysis of diarrheal stools made it possible to identify four (04) parasitic species: *Entamoeba histolytica* (15.64%), *Entamoeba coli* (6.70%), *Trichomonas intestinalis* (2.23%) and *Fasciola hepatica* (0.56%). These observations are consistent with those made in studies on parasites responsible for enteric infections in Nairobi, Kenya [30] and on pathogens responsible for diarrhea in Far North, Cameroon [14] where cysts of *E. histolytica* have been found at a higher frequency than other parasites in children. This could be explained by the endemic nature of *Entamoeba histolytica* and their widespread distribution in intertropical countries, associated to environmental factors, including lack of drinking water supply, lack of hygiene, and the level of household sanitation.

In this study, the major bacterial etiological agent was identified to be *Escherichia coli* (10.61%), *Salmonella spp*. (5.02%), *Klebsiella spp*. (3.35%), *Enterobacter cloacae* (1.12%), *Klebsiella pneumoniae* (1.12%), *Shigella spp.* (1.12%), *Enterobacter aerogenes* (0.56%), *Enterobacter spp*. (0.56%), *Hafnia alvei* (0.56%) and *Pseudomonas spp*. (0.56%). Many studies have also highlighted the role of *E. coli* as an ethiogical agent for diarrheal and inflammatory bowel diseases [33, 34]. This is consistent with the results obtained following a study on bacterial diarrhea in Nigeria where *Escherichia coli* was identified with the highest frequency among the causative agent responsible for diarrhea in children [35]. The observation was made following a study carried out in Burkina Faso [31], or following those carried out in Cameroon in the Littoral Region [36] and the Far North regions [14]. This could be explained by the endemic nature of *E. coli* and its widespread distribution in intertropical countries. Indeed, diarrhea is a water‐borne disease [37, 38] that can be caused by several environmental factors, including lack of drinking water supply, lack of hygiene, and the level of household sanitation [39, 40]. At least 2 billion people worldwide use a drinking water source contaminated by fecal matter [41]. In Cameroon, 85% of households do not treat drinking water and 49% of the population of Ngaoundere (Adamawa Region) does not have access to an appropriate water supply source [11]. Microbiological contamination of drinking water can cause the transmission of diarrheal diseases.

The fact that this study was conducted only on patients who came for treatment to the Ngaoundere Regional Hospital for a period of 4 months would constitute a limitation for this study and would influence the incidence of the disease. Self‐medication, the use of medicinal plants by the population in the treatment of diarrhea as well as the purchasing power of certain households would limit the number of patients who could consult hospital services [42]. It would be necessary in the future to extend this study to households and over a longer period.

## 5 Conclusion

This study aimed to determine the main infectious agents responsible for diarrhea in children under 5 years of age attending the Ngaoundere Regional Hospital between May 2023 and August 2023. It appears that diarrheal diseases affect all age groups (between 0 and 5 years) of population in the Ngaoundere city, with a higher prevalence in males children aged 12–23 months. Infectious diarrhea accounted for 72.06% of total cases of diarrhea in the population studied. Among the infectious agents, *Entamoeba histolytica* and *Escherichia coli* were the most incriminated in the occurrence of diarrhea. Clarification of the main microorganisms and data on their prevalence will be essential for the establishment of effective primary health care activities as well as for the prevention of diarrheal diseases.

## Author Contributions

**Michel Archange Fokam Tagne:** conceptualization, writing – original draft, investigation, writing – review and editing, methodology, software, formal analysis. **Benjamin Talom Tangue:** investigation, writing – original draft, writing – review and editing. **Emilie Laurence Kuete Diffo:** writing – review and editing, methodology, investigation, formal analysis, writing – original draft. **Paul Aimé Noubissi:** conceptualization, writing – original draft, writing – review and editing, formal analysis. **Angèle Foyet Fondjo:** investigation, validation, writing – original draft, writing – review and editing. **Yafet Dourkangou:** data curation, writing – review and editing, visualization. **René Kamgang:** conceptualization, writing – review and editing, supervision.

## Conflicts of Interest

The authors declare no conflicts of interest.

## Transparency Statement

The lead author, Michel Archange Fokam Tagne, affirms that this manuscript is an honest, accurate, and transparent account of the study being reported; that no important aspects of the study have been omitted; and that any discrepancies from the study as planned (and, if relevant, registered) have been explained.

## Data Availability

The data that support the findings of this study are available from the corresponding author upon reasonable request.
